# Spatio-Temporal Variability of Aquatic Vegetation in Taihu Lake over the Past 30 Years

**DOI:** 10.1371/journal.pone.0066365

**Published:** 2013-06-18

**Authors:** Dehua Zhao, Meiting Lv, Hao Jiang, Ying Cai, Delin Xu, Shuqing An

**Affiliations:** Department of Biological Science and Technology, Nanjing University, Nanjing, P R China; Catalan Institute for Water Research (ICRA), Spain

## Abstract

It is often difficult to track the spatio-temporal variability of vegetation distribution in lakes because of the technological limitations associated with mapping using traditional field surveys as well as the lack of a unified field survey protocol. Using a series of Landsat remote sensing images (i.e. MSS, TM and ETM+), we mapped the composition and distribution area of emergent, floating-leaf and submerged macrophytes in Taihu Lake, China, at approximate five-year intervals over the past 30 years in order to quantify the spatio-temporal dynamics of the aquatic vegetation. Our results indicated that the total area of aquatic vegetation increased from 187.5 km^2^ in 1981 to 485.0 km^2^ in 2005 and then suddenly decreased to 341.3 km^2^ in 2010. Similarly, submerged vegetation increased from 127.0 km^2^ in 1981 to 366.5 km^2^ in 2005, and then decreased to 163.3 km^2^. Floating-leaf vegetation increased continuously through the study period in both area occupied (12.9 km^2^ in 1981 to 146.2 km^2^ in 2010) and percentage of the total vegetation (6.88% in 1981 to 42.8% in 2010). In terms of spatial distribution, the aquatic vegetation in Taihu Lake has spread gradually from the East Bay to the surrounding areas. The proportion of vegetation in the East Bay relative to that in the entire lake has decreased continuously from 62.3% in 1981, to 31.1% in 2005 and then to 21.8% in 2010. Our findings have suggested that drastic changes have taken place over the past 30 years in the spatial pattern of aquatic vegetation as well as both its relative composition and the amount of area it occupies.

## Introduction

With the rapid development of the Chinese economy since the “reform and opening-up” policy was implemented in 1978, human activities have placed a growing stress on the country’s freshwater lakes, which are extremely vulnerable to natural and anthropogenic disturbance [1]–[3]. As a result, most freshwater lake ecosystems in China have experienced drastic changes during this period [4], [5]. Because of the important ecological and socioeconomic functions of aquatic macrophytes, such as stabilization of sediments, purification of water, slowing of water currents and maintenance of fishery production [6]–[10], examining the temporal dynamics of these organisms following implementation of the 1978 policy changes in China can provide valuable information concerning the mechanisms driving shifts in distribution of aquatic macrophytes that can be used to better manage inland waters.

Taihu Lake, the third-largest freshwater lake in China, is located in the core of the Yangtze Delta within the lower reaches of the Yangtze River Basin, one of the most developed areas in China. Since the 1978 policy changes, the Taihu Lake catchment has experienced rapid socio-economic development. Currently, the catchment contains 3.7% of the Chinese population and 11.6% of its Gross Domestic Product (GDP) within an area of 36,900 km^2^ that accounts for only 0.4% of China’s total land area. Concurrent with the rapid socio-economic development, the aquatic ecosystem of Taihu Lake has degraded appallingly [11]–[13], with the degradation being widely attributed to eutrophication and human activities such as flood control projects and wetland reclamation [11], and the distribution and community structure of aquatic macrophytes have clearly changed [14], [15].

Although several field inventories conducted since the 1960s have provided data on community structure in Taihu Lake, most of these inventories have provided little or only approximate information on distributional ranges due to technological limitations in addition to the substantial variability in distribution area both within a year and among years [14], [15]. Because continuous data sets containing information on exact distributions of different aquatic vegetation types are lacking, the ability to monitor the dynamics of aquatic vegetation and identify the driving forces behind changes in its distribution is restricted. Moreover, disturbances resulting from human activities [15], [16] inhibit the ability to clearly identify the particular role of eutrophication in the temporal succession process, further limiting our understanding of the forces driving succession.

We divided Taihu Lake into six sections according to the relative influences of human activities and environmental factors in order to better describe the spatio-temporal variability. By reconstructing the distribution of aquatic vegetation types in each of the six sections from 1981 to 2010 using a series of Landsat images (i.e. ETM+, TM and MSS images) and field validation inventories from 2009 and 2010, this study sought to track the spatio-temporal variability of aquatic vegetation distribution in Taihu Lake since the “reform and opening-up” policy was implemented in 1978.

## Materials and Methods

### 2.1 Study Area

Our study area included the entirety of Taihu Lake as well as the surrounding area within 500 meters of the lake boundary, where most of the emergent vegetation was distributed. Taihu Lake has an average depth of 1.9 m and occupies a surface area of 2,425 km^2^. The Taihu Lake catchment plays an important role in China’s political economy, with the GDP per capita in this area exceeding US$8,000 after 2007, more than three times the country’s average. Per unit of land, the catchment’s economic yield is 57 times the national average [11]. Indirect human activities such as flood control projects and pollution inputs, as well as direct human activities such as wetland reclamation and pen-fish-farming have transformed the formerly healthy aquatic ecosystem of Taihu Lake [16].

**Figure 1:**
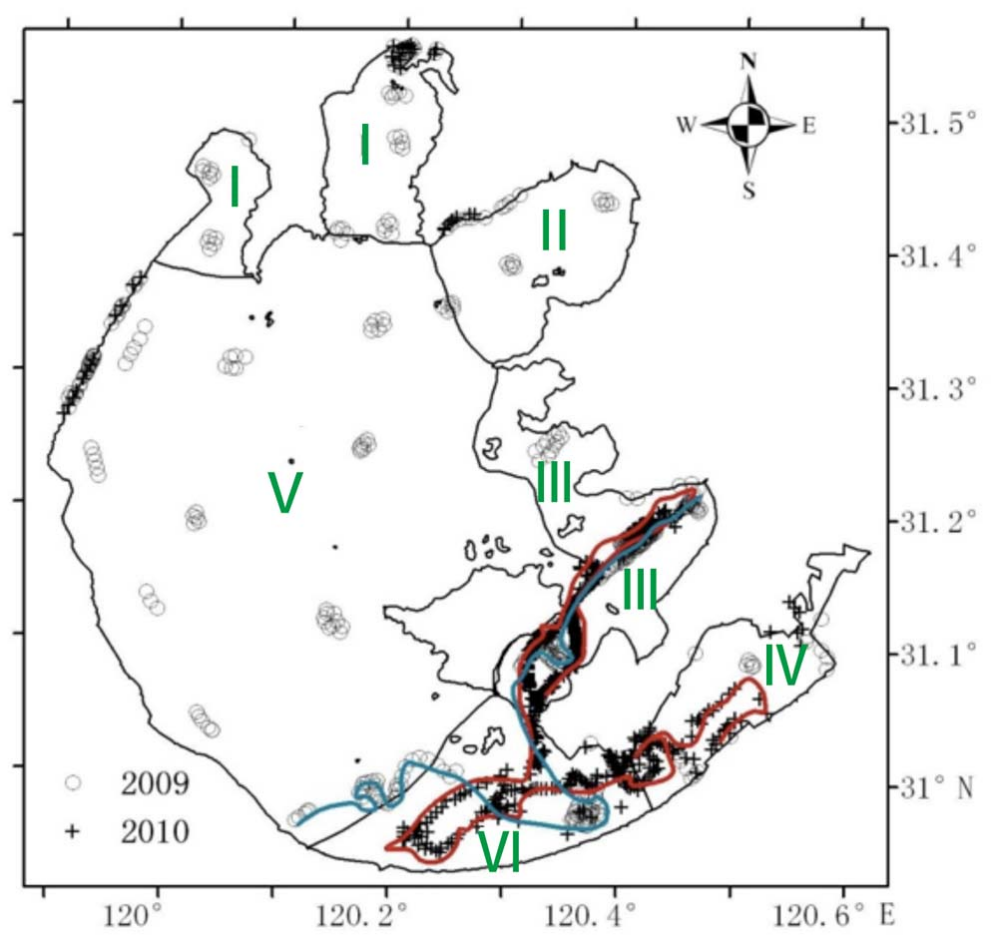
The study area showing the six sections of Taihu Lake and the distribution of 965 training samples (426 in 2009 and 539 in 2010) of emergent, floating-leaf and submerged macrophytes and other compositional types.

Because of the substantial spatial variation in the aquatic environments of Taihu Lake, we divided the lake into six sections (Fig. 1): I, Meiliang Bay and Zhushan Bay, the most polluted area in the lake [17]; II, Gonghu Bay, through which large amounts of water have been flushed into the lake from the Yangtze River since 2001 [18], [19]; III, the eastern coastal areas that represent the traditional distribution area of aquatic vegetation; IV, the east bay, where most of the activities related to fisheries production are focused; V, the central area and the west coastal areas, which occupy 58.8% of the lake; VI, the southeast section, which connects the central area and the east bay.

We used the spectral characteristics of the remotely-sensed images to classify the aquatic vegetation of Taihu Lake into three types: emergent vegetation, floating-leaf and floating vegetation, and submerged vegetation [20], [21]. To investigate the temporal dynamics of aquatic vegetation distribution in Taihu Lake over the past 30 years, we mapped the distributions at approximate five-year intervals. Considering the availability of clear remote sensing images, the final study years were chosen to be 1981, 1984, 1989, 1995, 2000, 2005, 2009 and 2010 (Table 1). The 2009 data were used to evaluate the method developed in this study to identify aquatic vegetation types.

**Table 1:** Dates of the Landsat ETM+, TM and MSS images used in this study.

Years	Sensors	Date-1	Date-2	Date-3
2010	ETM+	3/13	8/20	9/21
2009	TM	1/13	8/25	9/10
2005	ETM+	3/31	6/19	9/7
2000	ETM+	3/18	8/8	10/12
1995	TM	2/24	8/3	8/19
1989	TM	1/14	7/17	10/21
1984	MSS	12/16 (1983)	8/4	9/5
1981	MSS	2/4	7/16	9/8

(Due to the absence of high quality MSS images in the winter of 1984, the MSS image dated 16 December 1983 was used instead).

### 2.2 Field Surveys

Field surveys were conducted on 14–15 September 2009 and 27 September 2010 to gather data for model building and accuracy assessment of the classified maps, with 783 samples collected for open water or aquatic vegetation (Fig. 1). An additional 182 samples of reed (emergent) vegetation or terrestrial areas (e.g., shoreline roads and buildings such as docks, businesses and factories) were obtained from a 1∶50,000 land use and land cover map due to logistical difficulties in maneuvering a boat in the dense reed vegetation. A total of 426 and 539 training or validation samples were collected for 2009 and 2010, respectively. The field survey procedures have been described in detail by Zhao et al. [20].

### 2.3 Image Processing

The procedure used in this study was similar to that implemented by Zhao et al. [20] and thus is only briefly summarized here. Images used in this study had no more than 10% cloud cover in accordance with the recommended standard for aquatic remote sensing [22]. Prior to atmospheric correction, cloud-contaminated pixels were removed from all images using interactive interpretation. Atmospheric corrections were applied to the images using the cosine approximation model (COST; Chavez, 1996) using ERDAS IMAGINE 9.2 (Leica Geosystems Geospatial Imaging, LIC), and geometric correction was carried out for all the images using second-order polynomials with an accuracy higher than 0.5 pixel.

We used combinations of winter (January through March when the biomass of aquatic vegetation was lowest) and summer (June through October when the biomass of aquatic vegetation was highest) images from each study year for aquatic vegetation identification. For each study year, three clear Landsat images were selected (one from winter and two from summer) and formed into two pairs in which the winter image was paired with each summer image. Thus, a total of sixteen pairs were used in this study (Table 1). The ground truth samples from 2009 and 2010 were used to evaluate the accuracy of the final aquatic vegetation classifications derived from the 2009 and 2010 image pairs.

### 2.4 Aquatic Vegetation Identification

To identify emergent, floating-leaf and submerged vegetation, we developed classification tree (CT) models for direct application between images from different dates and sensors [20], [21]. Considering the differences in both wavelength range and the spectral response curve among images from different sensors (i.e., ETM+, TM and MSS), we developed classification model structures manually for emergent, floating-leaf and submerged vegetation and then obtained quantitative thresholds for specific images from CT analysis of the data in our statistical software package (PASW-Statistics v. 18). We selected three spectral indices to identify emergent, floating-leaf and submerged vegetation: the Normalized Difference Vegetation Index (NDVI) [23], Modified Normalized Difference Water Index (MNDWI) [24], and average reflectance of the blue, green and red bands from the remote sensing image (AVE123) [25]. Summer (−s) and winter (−w) values, as well as the difference between the summer and winter values (−s−w), were calculated for each of these indices for use in the models.

**Figure 2:**
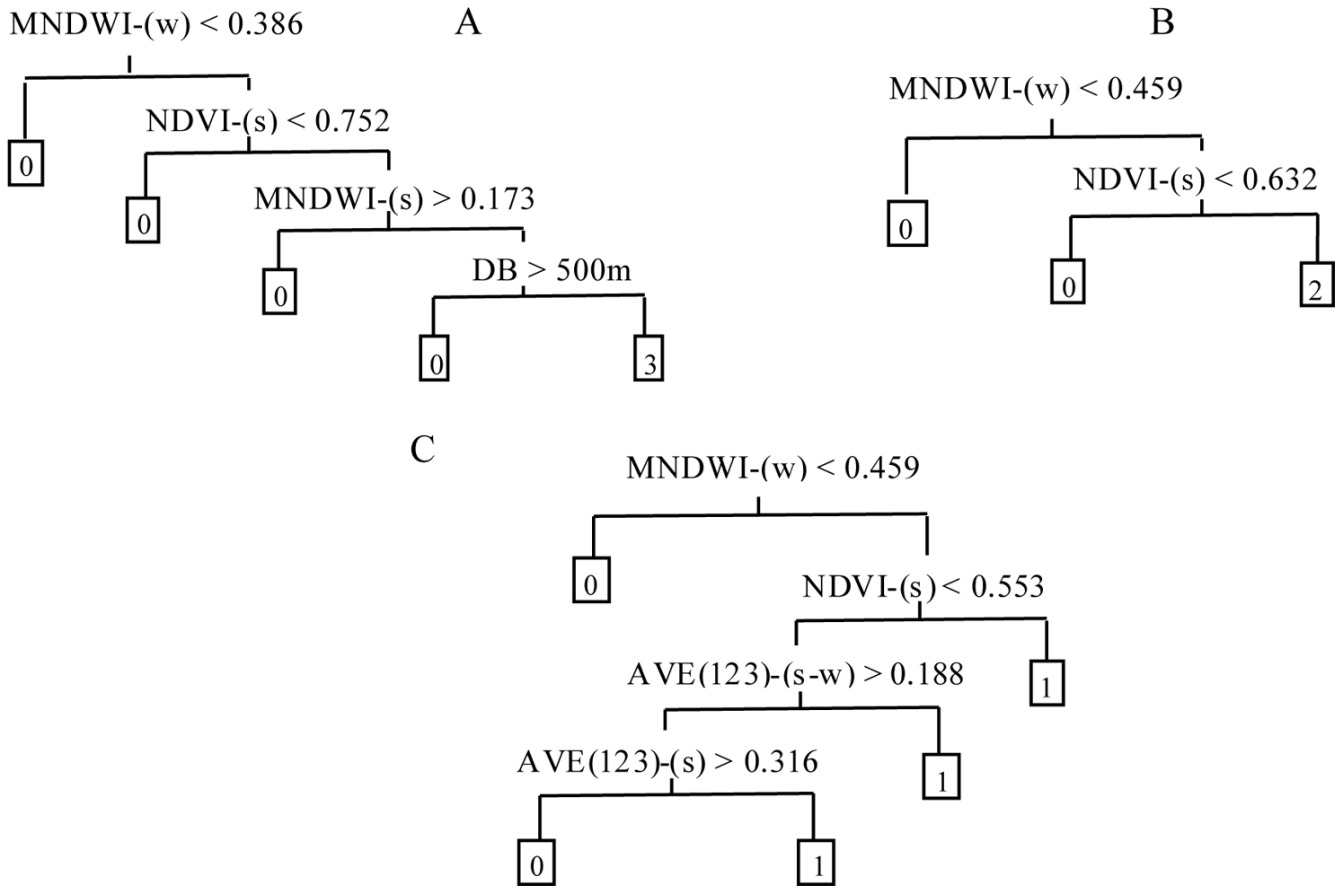
Classification tree models established for (A) emergent vegetation, (B) floating-leaf vegetation and (C) submerged vegetation. The numbers 3, 2 and 1 in the end nodes of the classification trees represent emergent, floating-leaf and submerged vegetation, respectively, whereas 0 represents other types. Variables used are the Modified Normalized Difference Water Index (MNDWI), the Normalized Difference Vegetation Index (NDVI), the average reflectance of the blue, green and red image bands (AVE123) and the distance to the lake bank (DB). Variables were calculated by season (s = summer, w = winter) or differences among seasonal values (e.g., s-w).

The basic classification model structures for identification of emergent, floating-leaf and submerged vegetation are shown in Fig. 2. Three spectral indices, MNDWI-(w), NDVI-(s) and NDWIF-(s), and one spatial parameter, distance to boundary (DB), were used for identification of emergent vegetation; two spectral indices, MNDWI-(w) and NDVI-(s), were used to identify floating-leaf vegetation; and four spectral indices, MNDWI-(w), NDVI-(s), AVE123-(s-w) and AVE-(s), were used to identify submerged vegetation.

### 2.5 Robustness of CT Models

CT models developed for 2009 image pairs (Fig. 2) had an overall accuracy of 92.5%, with classification accuracies of 89.1%, 92.9% and 88.8% for emergent, floating-leaf and submerged vegetation, respectively. When the CT models were applied to the image pairs of 2010, overall accuracy was 91.7%, with classification accuracies of 91.4%, 89.2% and 87.9% for emergent, floating-leaf and submerged vegetation, respectively (Table 2). These results suggested that the CT analysis could be used to effectively identify the aquatic vegetation in Taihu Lake. Therefore, the CT models were used to map the distribution of aquatic vegetation at different times during the past 30 years.

**Table 2:** Confusion matrix of the CT models developed in this paper as applied to 2009 and 2010 data, respectively (in number of field samples).

			Prediction					
			Emergentvegetation	Floating-leafvegetation	Submergedvegetation	Other types	Classificationaccuracy (%)	Overall accuracy (%)
**2009**	**Truth**	**Emergent vegetation**	49	4	1	1	89.1	92.5
		**Floating-leaf vegetation**	4	91	3	0	92.9	
		**Submerged vegetation**	0	5	103	8	88.8	
		**Other types**	0	1	5	151	96.2	
**2010**	**Truth**	**Emergent vegetation**	74	7	0	0	91.4	91.7
		**Floating-leaf vegetation**	5	132	7	4	89.2	
		**Submerged vegetation**	0	6	102	8	87.9	
		**Other types**	0	0	8	186	95.9	

## Results

### 3.1 Spatio-temporal Dynamics of Distribution Area

**Figure 3:**
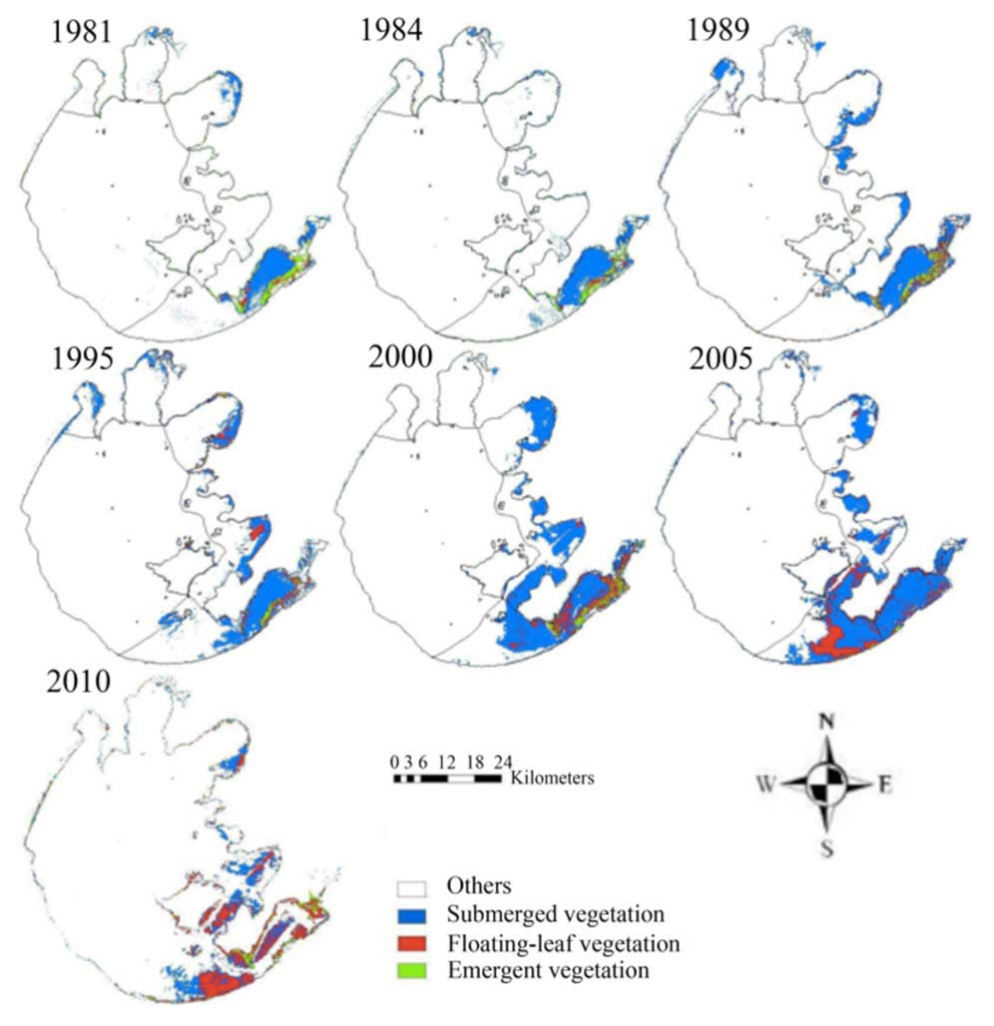
Maps showing the distribution of emergent, floating-leaf and submerged vegetation in Taihu Lake in 1981, 1984, 1989, 1995, 2000, 2005 and 2010.

**Figure 4:**
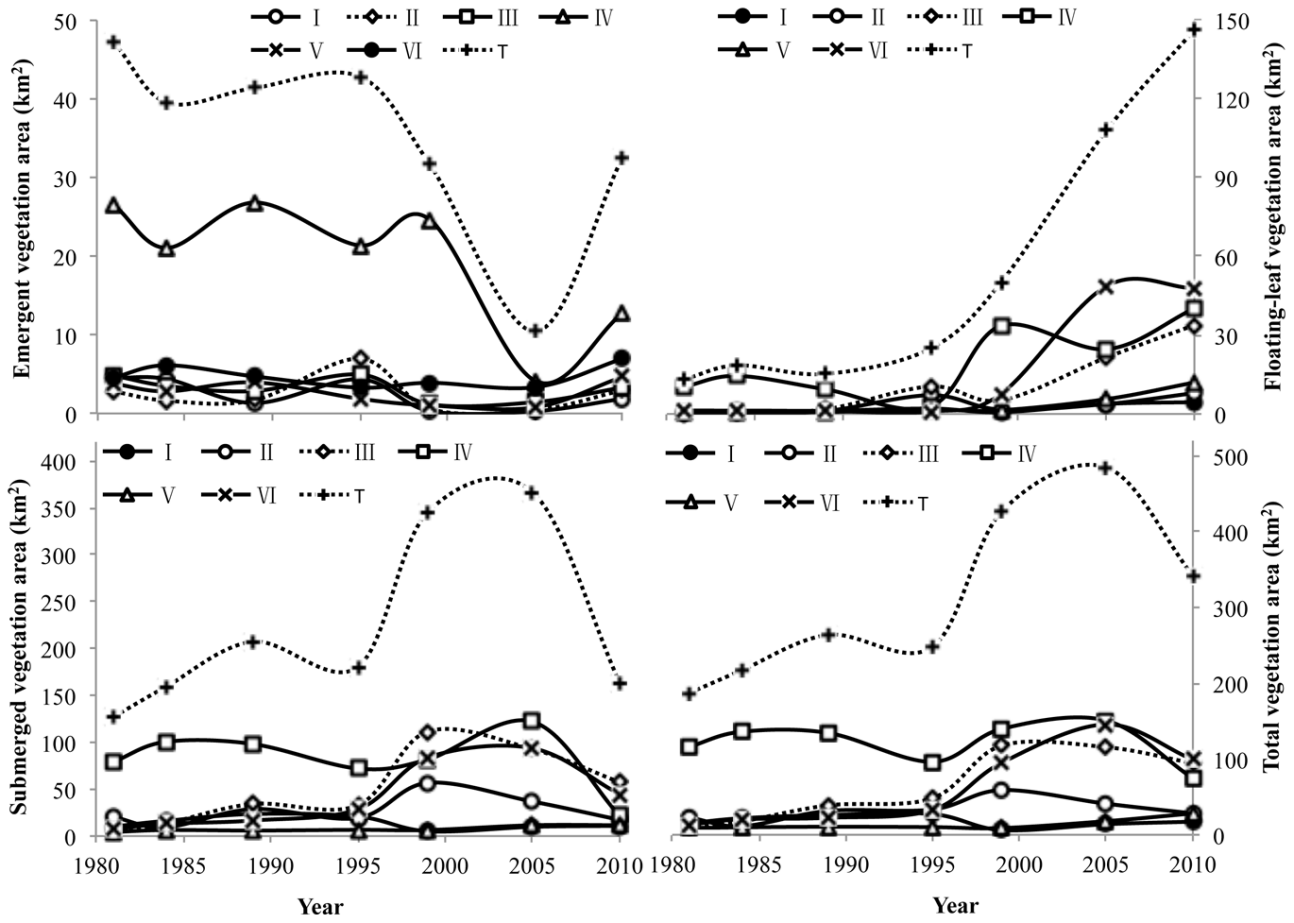
The temporal dynamics of emergent, floating-leaf, and submerged vegetation as well as those of total aquatic vegetation area (the sum of emergent, floating-leaf, submerged vegetation) in the six sections of Taihu Lake (see Fig. 1) between 1981 and 2010. T is the total area of the lake (i.e. the sum of the six sections).

We used the CT models developed in this study to map emergent, floating-leaf and submerged vegetation in 1981, 1984, 1989, 1995, 2000, 2005 and 2010 (Fig. 3). Substantial changes in aquatic vegetation distribution have taken place over the past 30 years. From 1981 to 2005, the area of aquatic vegetation gradually increased, from 187.5 km^2^ in 1981 to 485.0 km^2^ in 2005– a 159% increase (Fig. 4). However, compared with 2005, the area of aquatic vegetation decreased suddenly in 2010 to 341.3 km^2^.

In addition to distribution area, the spatial pattern of aquatic vegetation has also experienced substantial temporal variation. Over the past 30 years, aquatic vegetation has spread gradually from the East Bay (section IV) to sections III and VI. In section IV, aquatic vegetation area increased from 116.6 km^2^ in 1981 to 150.7 km^2^ in 2005 and then suddenly decreased to 74.5 km^2^ in 2010, but the proportion of vegetation in section IV relative to that in the entire lake decreased continuously from 62.3% in 1981, to 31.1% in 2005 and then to 21.8% in 2010. In sections III and VI, aquatic vegetation area increased, respectively, from 10.1 and 13. 3 km^2^ in 1981 (5.4% and 8.3% of the total area) to 115.7 and 145.6 km^2^ in 2005 (23.9% and 30.0% of the total area), and then decreased to 94.5 and 99.2 km^2^ in 2010 (27.6% and 29.0% of the total area).

### 3.2 Composition of Emergent, Floating-leaf and Submerged Vegetation

Drastic changes have occurred to the areas occupied by all the vegetation types (emergent, floating-leaf and submerged) over the past 30 years, and the temporal dynamics differed by vegetation type (Fig.4). The area occupied by emergent vegetation gradually decreased from 47.7 km^2^ in 1981 to 10.6 km^2^ in 2005 (a 77.8% decrease) and then increased to 31.8 km^2^ in 2010 (a 300% increase over 2005). The temporal dynamics of emergent vegetation in section IV followed this pattern, decreasing from 26.6 km^2^ in 1981 to 4.2 km^2^ in 2005 and then increasing to 12.8 km^2^ in 2010. The area occupied by floating-leaf vegetation increased continually through time, from 12.9 km^2^ in 1981 to 146.2 km^2^ in 2010, a 10.3-fold increase. This continuous increase was primarily a result of the increases in sections III, IV and VI, where floating-leaf vegetation increased 33.6, 29.6 and 46.5 km^2^, respectively, in 2010 over that in 1981. Submerged vegetation, on the other hand, gradually increased in area from 127.0 km^2^ in 1981 to 366.5 km^2^ in 2005 (a 189% increase), then decreased suddenly in 2010 (163.3 km^2^).

Substantial changes were also observed in the spatial pattern for each vegetation type. In 1981, emergent, floating-leaf and submerged vegetation were primarily distributed in section IV, where they comprised 56.3%, 81.2% and 62.6%, respectively, of the total area occupied by these vegetation types within the entire lake. After 1981, emergent, floating-leaf and submerged vegetation gradually expanded outward from section IV to the adjacent sections. By 2010, the proportion of emergent, floating-leaf and submerged vegetation in section IV had decreased to 39.2%, 27.5% and 13.2%, respectively, of the total area occupied by these types.

Additionally, the relative abundances of the three aquatic vegetation types changed substantially over the 30 years of this study. Emergent vegetation decreased from 25.4% of total aquatic vegetation in 1981 to only 2.18% in 2005, then rose slightly to 9.31% in 2010; this was consistent with the emergent vegetation in section IV, which decreased from 22.9% of total aquatic vegetation within this section in 1981 to 2.76% in 2005, then rose slightly to 17.2% in 2010. Floating-leaf vegetation increased as a percentage of total aquatic vegetation over the study period, from 6.88% in 1981 to 22.3% in 2005 and then to 42.8% in 2010. The percentage of submerged vegetation increased from 67.7% in 1981 to 75.6% in 2005 and then decreased to 47.9% in 2010.

## Discussion

### 4.1 Driving Forces

The high spatio-temporal variability of aquatic macrophyte distribution in Taihu Lake over the past 30 years can be attributed to both direct and indirect influences of human activities [11]. Because consistency is lacking with regard to quantitative information on the spatio-temporal vegetation dynamics in relation to human activities, we are limited to a qualitative discussion of the forces driving the observed changes.

#### 4.1.1 Direct influences of human activities

**Figure 5:**
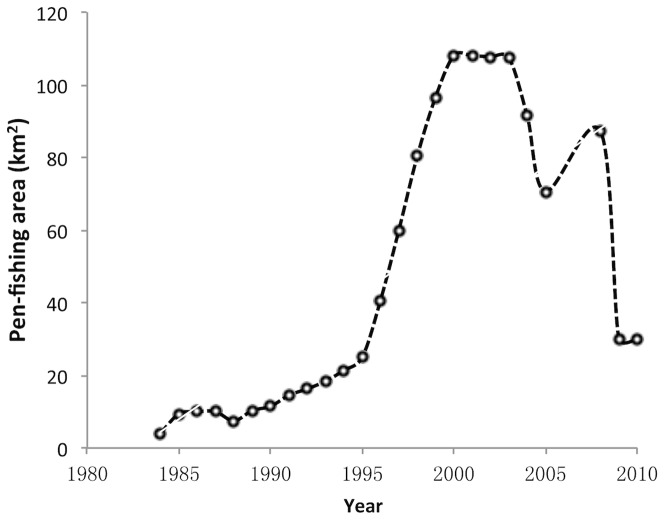
Temporal dynamics of area open to pen-fishing in Taihu Lake in the past 30 years.

Human activities such as planting and conservation or harvesting and removal have affected the distribution area of aquatic vegetation directly. The incentives for planting and conserving aquatic vegetation in the lake came primarily from fishery production activities that can be quantified by pen-fish area. Pen-fish farming activities in Taihu Lake began in the early 1980s and reached a peak in 2000 (108.1 km^2^, accounting for more than 80% of the East Bay of Taihu Lake), after which the pen-fishing area began to decrease (Fig. 5). In 2009 and 2010, the area open to pen-fishing decreased suddenly to only about one-fourth what it was in 2000.

In addition to pen-fishing activities, numerous water conservation projects in recent years have slightly increased the lake area occupied by vegetation through planting and restoration of aquatic macrophytes [26]. Direct removal of aquatic vegetation in Taihu Lake has resulted primarily from four types of human activities: (1) wetland reclamation for farming and building construction, which took place mostly prior to 1985 [14], [15]; (2) flood-control projects that built concrete embankments instead of maintaining the original wetland buffer zones [11]; (3) large-scale dredging that was implemented in section IV and the surrounding area in recent years [26], [27]; and (4) harvesting of aquatic vegetation to be used as food for fish. These direct influences probably had the greatest impact on the distribution of aquatic vegetation in section IV as well as the distribution of emergent vegetation within the entire lake [28].

#### 4.1.2 Indirect influences of human activities

Indirect impacts of human activities on the aquatic vegetation of Taihu Lake were more complex than direct planting or removal activities. First, the high sedimentation rate and swampy conditions in section IV and the area surrounding section VI were likely responsible for the increase in distribution area of aquatic vegetation in section VI before 2005, which was mitigated by the implementation of dredging after 2007 [14], [28], [29]. Second, water level and its seasonal pattern, determined largely by flood-control projects and artificial regulation, influenced the distribution area and composition of aquatic vegetation in the entire lake [14], [15], [30]. Third, eutrophication caused primarily by exogenous input from surrounding rivers and fishery production activities probably acted as one of the most important factors affecting the temporal variability of both area and composition of aquatic vegetation in sections I, III and V [15], [31], [32]. Fourth, the action of flushing water into the lake from the Yangtze River through section II decreased water clarity and thus decreased submerged vegetation in section II [18], [19], [33].

### 4.2 Implications for Management

Currently, eutrophication is one of the main aquatic environmental problems in Taihu Lake [34]. In order to remove excess nutrients and recover the degraded aquatic ecosystem of Taihu Lake, numerous costly water conservation projects that include planting and restoration of aquatic macrophytes have been implemented in recent years, particularly after the water supply crisis in Wuxi City that was induced by the 2007 blue-green algal bloom [11], [35], [36]. Our results indicated that aquatic macrophytes are distributed over a large area in Taihu Lake and experienced substantial changes in their distributions over the past 30 years (i.e. between 187.5 and 485.0 km^2^). Compared to the expensive water conservation projects, it is probably more economical to protect existing communities of aquatic macrophytes [30]. Therefore, future study should be focused on the effective management of the hundreds of square kilometers of aquatic macrophytes in Taihu Lake. However, we cannot over-rely on the purification function of aquatic vegetation for the recovery of the aquatic ecosystems in Taihu Lake because the aquatic ecosystem can shift suddenly from a clear-water plant-dominated state to a turbid algal-dominated state if the pollutants increase further in the lake [37]. Because it is much more difficult to promote a shift in aquatic ecosystems from the turbid algal-dominated state to the clear-water plant-dominated state than it is to instigate a shift in the opposite direction [37]–[39], preventing the initial shift from the clear-water plant-dominated state to the turbid algal-dominated state is probably one of the most important current goals for effective lake management.

Due to the different ecological and socioeconomic functions performed by different species and the different aquatic vegetation types such as submerged and floating-leaf vegetation [14], [40], the artificial regulation of aquatic vegetation composition is also very important for the management of Taihu Lake. Field studies have indicated that the dominant species in the lake have changed from *Potamogeton maackianus*, *Hydrilla verticillata*, *Vallisneria spiralis* and *Zizania caduiftora*, to *Elodea muttalli*, *Potamogeton malaianus* and *Nymphoides peltata*. The area covered by *Zizania caduiftora* and *Phragmites communis* has decreased drastically in response to human activities - plantation reclamation in particular [14], [15], [40]. Our findings were consistent with these field investigations: the area of emergent vegetation gradually decreased, while the ratio of floating-leaf vegetation to submerged vegetation gradually increased over the past 30 years. Current management strategies in Taihu Lake such as the large-scale dredging carried out in section IV and the surrounding area after 2007 [28], [29], the decrease in eutrophication [31] and the decrease in pen-fishing area [32] are probably beneficial to restoring Taihu Lake to its original submerged-dominant status, whereas water level management activities that decrease the water level in the rainy season and increase the water level in the dry season will likely result in the opposite scenario [30].
